# The Casket and the Ribbon, or the Honors of Ether

**Published:** 1849-07

**Authors:** William H. Dwinelle


THE AMERICAN

### Journal of Dental Science.

Vol. IX.] JULY, 1849. [No. 4.

### ARTICLE I.

### *The Casket and the Ribbon, or the Honors of Ether.* By WILLIAM H. DWINELLE, M. D., D. D. S.

Who has ever witnessed that great miracle of modern science—a painless surgical operation, without feeling inclined to exclaim, with Lord Byron,

> "Oh! thou beautiful
> And unimaginable *Ether!*"

THE transcendent value and importance, indeed, of the ether discovery is universally recognised. It is sufficiently proved by the bitter and protracted controversy which has arisen between its rival claimants. That controversy is now substantially ended.

The masterly report of the Committee of Congress, presented by the Hon. Thomas O. Edwards, M. D.—its clear and simple statements of the question at issue—its searching analysis of the evidence—its striking illustrations—its conclusive and logical deductions, have, as we believe, convinced all, except those few partizans of Dr. Jackson, who, from personal friendship, professional bias, or the natural reluctance to abandon an early and cherished opinion, still adhere to his cause with unwavering fidelity. Without any parade of learning or scientific research, this document sets forth certain acts of the parties, and then by the plainest and most cogent arguments, irresistibly leads the reader to infer the motives and views of the actors. Without any severity of language, it quietly sets aside the false pretenses which came under its notice. Thus, Dr. Jackson claims that he made his discovery in 1842. If, however, he had the least realizing sense of this great truth, (it is agreed,) then he must have known, that immortal honor awaited its disclosure. He hears around him the cries of suffering—he is admitted to be an eager aspirant for fame—and no one doubts his kindly disposition. But he remains torpid for four years—deaf alike to the call of ambition and even to the dictates of common humanity. The unavoidable inference is, that he could not have had any strong, clear convictions in the case. We accede, therefore, at once, to the conclusion, that he merely had arrived at an induction or hypothesis, on the subject which he thought of little or no value, (probably as tending only to a slight improvement in *dental* surgery,) and thus entirely omitted to take any step to verify it.

Again, Dr. Jackson claims, that, at last, Dr. Morton, performed his experiments, as *his* agent—being the mere "nurse who adminstered *his* prescription." This claim, likewise, is shown to be surrounded by insuperable difficulties. Dr. Jackson, in an interview, *not sought by himself*, makes a mere casual suggestion to Dr. Morton—one whom he represents as grossly ignorant and reckless—to whom he refuses to give a written certificate of the safety of the application, and from whom he thenceforward holds himself wholly aloof. He is not present at the early experiments. He publicly denounces Dr. Morton as likely to kill somebody, yet, before he is done—expressing in the strongest manner, his regret that he had ever given him any information on the subject, &c.

Now, the inference of the Committee seems absolutely unavoidable, that Dr. Jackson, knowing, as he must have done, the importance of these first, test experiments—what science, skill and caution were necessary for their safety and success—could not have selected as his agent, such a man as his own witnesses represent Dr. Morton to be; and that, having selected him, he could not thus have conducted himself, throughout the series of these experiments.

Indeed, the deliberate claim, by Dr. Jackson, that these experiments were *his*—performed by *his* agent, and in *his* behalf, seems to us the act of a man, who, shunning all responsibility, during the period of danger and uncertainty, seeks, at last, to snatch away the prize which had been fairly won, by the labors and services of another.

The legal acumen shown by the Committee, is remarkable; it would do high honor to the most eminent practitioner. The fact that Dr. Morton's ignorance, (as manifested at his interview with Dr. Jackson,) was assumed, seems certain, from his having previously learned from Mr. Metcalf, the general properties of ether. The Committee, however, discover one circumstance, in confirmation of this position, which had before been wholly unnoticed, viz. that Dr. J. directed ether to be *spattered on a handkerchief;* thus really telling *that it was a liquid*—so that when Dr. M. in reply, asked *if it were a gas*, he must have been concealing what he knew.

The comparison of the testimony of Barnes and McIntyre is also most able and satisfactory. Dr. Keep and Don Pedro Wilson, are placed in an interesting contrast—without a word, however, charging either with intentional falsehood: and Dr. Keep's affidavit, that a certain paper contained a certain statement in favor of Dr. Jackson, is amusingly nullified by the production of the paper itself, containing nothing of the sort.

This report is brief, pertinent, searching, decisive, encumbered with no array of documents, and no irrelevant opinions or certificates. It adopts the four propositions contained in the Report of the Trustees of the Massachusetts General Hospital. It closes with a stricture upon the *"professional irregularity"* of which one of the Messrs. Lord, the attorneys of Dr. Jackson, had been guilty.

We repeat, that we consider that this document has brought to an untimely end the claim of Dr. Jackson, as the discoverer and first applier of etherization.

A minority Report was subsequently presented by two members of the Committee of Congress, which is certainly a more plausible statement, in Dr. Jackson's behalf, than had before appeared. It does not, however, as we conceive, disprove or even weaken the conclusions of the prior report, from which it dissents. It sets forth what no one has ever denied—that Dr. Jackson communicated to Dr. Morton *an induction* which he had made, viz. that ether could be used with safety and effect, during a dental operation.

The Committee next proceed with a long array of opinions of different individuals, as to Dr. Jackson's merit in having made this suggestion. President Everett and other remonstrants, (among whom, we regret to learn, are to be found many of the Boston dentists,) give all the credit to Jackson. Mr. Everett, as will be remembered, had originally been induced to ask

Dr. Jackson to give the public an account of *his* discovery. Next appears a letter from Hon. Franklin Dexter, of Boston, of the like tenor. Regarding him as a lawyer of great soundness and ability, we should be glad to know, whether, after reading the majority Report, his views remain unaltered. We are next favored with no less than three notes from Dr. Walter Channing, of Boston; the first signed with his initials only, the second with his name, and the last with the addition of his titles, "M. D. Professor." He mentions, in a postcript to the first, that he had actually written a large volume on the subject, and the object of the last seems to be merely to charge upon Dr. Jacob Bigelow, great ignorance or negligence, in never having read a certain passage in a work of Pereira, which had for some years been lying on his table—and to excite sympathy for Dr. Jackson as having been charged in the Hospital Report, with *theft,* in announcing, as the result of his own inferences and investigations, the use of ether, in 1841, under the same circumstances in which Pereira's work had prescribed its use in 1839. Dr. Channing begins by a misstatement of fact, which the Committee suffer to pass uncontradicted.

He speaks of Jackson as using ether in 1841—"*the very year,*" observes he, "*in which it is said that author alluded to its use;*"—and he says, "there is not the least reason to suppose, for a moment, that Dr. Jackson had ever seen or heard of Pereira's work." Now, the Hospital Report merely suggests, that Dr. Jackson, as a learned chemist, was doubtless familiar with the book in question—and, as he in fact did in 1841 only what Pereira had directed to be done in 1839, the Report proceeds to consider him as having discovered nothing on the subject which had not been in print before. It may be observed, by the way, that if *he* never did see the book, it is in evidence, that it was purchased by Dr. Morton. Neither Dr. Bigelow's reputation nor Dr. Morton's will, as we believe, suffer much from these epistles.

The next communication is from Dr. Luther V. Bell, to the effect, that if it had not been for Dr. Jackson "the world would still remain without this greatest blessing." Now, the Hospital Report distinctly recognized the value of Dr. Jackson's suggestion to Morton, and speaks of it as one "without which he would not have made the discovery, at that precise time, and *might have failed to do so at any time.*" Dr. Bell expresses also the opinion, that, as Dr. Jackson had made the like suggestion so repeatedly, for four years, the discovery would, at last, have came out, even if Dr. Morton had never existed.

If Dr. Bell were to-morrow to be obliged to submit to an amputation, we think he would be induced to ascribe a somewhat greater degree of importance to the *present* and *immediate enjoyment of this discovery,* through Dr. Morton's agency: and would think less favorably of the chance of its ultimate attainment without his aid. Dr. Bell, we understand, needs no titles of office, and has the good taste to append none to his signature.

We find letters from Drs. J. B. S. Jackson and John D. Fisher, strongly in Dr. Jackson's favor. Both are physicians of the Massachusetts General Hospital—which title the latter appends to his signature.

Next, comes a characteristic letter from Mr. Bowen, (editor of the N. A. Review,) in which he speaks of

*"the dentist* Morton :" making the same captious remarks, in the same flippant style, as in his recent editorial article, *on the defrauded orphans of Mr. Girard,* on past management, present condition, and future prospects of Harvard College and the Boston Athenæum.* Then came letters from Drs. Hare and Gibson, stating their impressions, in Dr. J's favor.

A letter from Mr. Prescott, the historian, with more discrimination, awards to him the suggestion : but to Dr. Morton, *"a share, and no mean one,"* in the discovery : viz. its verification. A letter from Charles G. Loring, Esq., one of Dr. Jackson's counsel, of course, takes the side of his client. It is not an uncommon circumstance for gentlemen of the legal profession to think favorably of their own cases—to regard their own geese as swans.

Having duly paraded these letters in behalf of Dr. Jackson, the committee next proceed to introduce others highly complimentary to Dr. Morton : which, however, (as they would make it appear,) refer merely to his meritorious services "in demonstrating the practical value of the discovery, and in contributing, perhaps ! more than any other person, towards its introduction into general use." It is certainly evidence

* Upon the principle that our convictions are not matters of volition, but the effect of fair evidence, both external and internal, Mr. Bowen, by the disingenuousness and ill humor displayed throughout the whole of his letter, has very unkindly placed it out of our power to believe him ; and were he an hundred times the editor of the North American Review, and wrote such letters, we should still remain in the same dilemma.

He evidently feels very bitter and unforgiving towards "Mr. Morton" for having ventured out of his proper sphere in treading upon the forbidden ground of scientific investigation. His woe and his anathema are upon him : both of which, however, we are happy to state, he has thus far been able to survive.

of great ingenuity on the part of the Committee, in this manner, to qualify and fritter away the most absolute and unequivocal recognitions of Dr. Morton's claims. Dr. Jackson can never despair, if his friends can find any ground for his pretensions left in such a letter as the following, from Dr. John Jeffries, of Boston, to Mr. Speaker Winthrop:

"Boston, January 10, 1849. Dear Sir,—Mr. Morton, who visits Washington, to seek some remuneration from government, for the benefit which he has conferred on the country, by the introduction of sulphuric ether, requests me to express to you my opinion: which I do most unreservedly—that the world is indebted *entirely* to Mr. Morton for the introduction of this agent, to produce insensibility to pain; and that it is a physical blessing not second to any that has been conferred upon suffering humanity," &c.

The Committee dispose of similar letters from his excellency, Gov. Briggs and ex-Gov. Morton, of Massachusetts, and Hon. John P. Bigelow, Mayor of Boston, by suggesting, that, as the writers used general terms, they probably rely more upon information from others, than upon any investigation of their own. The whole of these opinions, pro and con, have nothing to do with the subject. They *change* no *fact* and they *prove* no *fact* in the case.

After some dozen pages of the report have been thus occupied, the Committee next proceed to comment upon the character and competency of the Board of Trustees of the Hospital. It is announced as a fact, that there are few or no legal or scientific men among the members of that board; and they courteously suggest that the Trustees have shown partiality towards Dr. Morton, caused, doubtless, by ***a thirst for distinction***, and a wish to identify their institution with the discovery, by ascribing the chief merit to its verification.

Now three, at least, of that Board are on the list of counsellors at law, in Boston: and the chairman of the Committee for drafting the Report, has for twenty years been extensively engaged in a branch of that profession. Three, or more, are members of the American Academy of Arts and Sciences; one of them (Mr. J. A. Lowell) being, by his position as sole Trustee of the Lowell Institute, in Boston, brought more intimately into contact with scientific men and matters than almost any one in that community. He is one of the corporation of Harvard College; and three-quarters of the board are graduates of that institution. It is needless to add, that a more fair, intelligent or competent jury was probably never impanneled to try an issue. It is the duty of this Board annually to lay before the Corporation, a statement of the affairs of the Hospital, during the past year. What should have been done on this occasion? The greatest public service ever rendered by the institution has been performed; shall it be passed over unmentioned? Certainly not. Shall the mere naked fact be stated, that these experiments were performed at the request of Dr. Morton, and all mention, even of Dr. Jackson's name, be omitted? This would have been the simplest, perhaps the wisest, course. But would it have been any more satisfactory to Dr. Jackson and his friends? The course actually taken was, no doubt, that by which the Committee conscientiously endeavored to do full and equal justice both to "the Dentist Morton," whom they had never seen, and to Dr Jackson, their old acquaintance and friend.

So obvious was this wish on their part, that the chief medical review of the county, (Hays',) for that very reason, reprinted the Report verbatim.

It is certainly difficult to imagine any possible bias or interest, on the part either of the Institution or the Trustees, to the *prejudice* of Dr. Jackson. Personal regard—his position in society—his standing as a man of science—would all seem to give him great advantages over his opponent. Dr. Jackson, indeed, very modestly suggested to the Committee of the Hospital, that a partiality was felt towards Dr. Morton, under the idea, that if the claims of one so ignorant were eulogized, rather than his own, *the institution would thereby acquire a larger share of credit as accoucheurs of the discovery.* What possible bearing, however, can it have in the case, how Dr. Morton happened to be led to think of the subject? The operations were actually performed at *his* sole request, by surgeons who had never heard of Dr. Jackson's name, in connection of the discovery. This is a "fixed fact." How is it altered or effected by the subsequent information, that Dr. Morton acted pursuant to a suggestion of Dr. Jackson? They had performed the operations, on their own responsibility—unaided by a word of advice or caution from Dr. J.—and the credit of the "delivery"—be it more or less—belongs to them, no matter who proves to be the father of the child.

To explain the position of the Massachusetts General Hospital the Committee introduce two letters. The first is from Dr. W. J. Walker, an eminent surgeon, of Boston, or its vicinity, now retired from practice. It is believed that Dr. Jackson at one time, had no great reason to rely on that gentleman's support, since we are informed that he has heretofore expressed the opinion "that the whole thing was Wells'—that neither Morton nor Jackson had any claim to it."

The second letter is from L. M. Sargent, Esq., well known as author of the Temperance Tales. This epistle is the gem of the whole collection. Mr. Sargent is a ready, playful and caustic writer, and none can fail to be amused with this production, which exhibits, in a strong light, all his peculiarities. We have no doubt that Mr. Bowditch laughed as heartily as any one at the paragraph about the casket (containing $1000) given to Dr. Morton, at Mr. B's suggestion: and the quiet humor with which he ascribes to that gentleman the power of extracting $10 a piece out of every body, for any object which he is willing to indorse. Dr. J. is represented as pointing out a hidden treasure, which Morton accordingly digs for and finds; but not a word is said of "the generally received opinion of the whole neighborhood," that there was a deadly snake concealed near it, who had so long proved an efficient guardian of the treasure.

As a specimen of the caustic, we select the following sentence: "It appears to us, that no impartial man of good common sense can read the evidence from the time of the demijohn to that of the casket, and hesitate to reject the claims of Dr. Morton as without any other foundation than stubble and rottenness." This last word might have been fitly used by "The sexton of the old school."* The letter concludes with a specimen of logic. The Hospital Report, it will be remembered, speaks of the discovery as consisting in the establish-

* A signature adapted in a series of newspaper articles published in the Boston Transcript.

ment or actual proof of the hypothesis, by direct experiment. "By so doing," (i. e. pulling out a tooth,) say the Trustees, "he made this discovery." In regard to all prior steps, the Report states *negatively*, that Dr. Jackson did not appear to have discovered any thing new, &c. Mr. Sargent thinks this required "a great deal of knowledge, or a great deal of presumption." But it certainly required nothing of either, to state affirmatively, that a tooth had been pulled out, and a denial of *Morton's* claims (one would suppose) involves as much presumption, or requires as much knowledge as a denial of *Jackson's*. The writer only ventures, in conclusion, to observe that his *impressions*—he will not say *convictions*—are decidedly in favor of Dr. Jackson. Letters from Dr. Jacob Bigelow and Dr. George Hayward, *fully indorsing the Hospital Report*, close this list. These gentlemen are disposed of by the remark, that they are probably the persons alluded to in Dr. Walker's letter. The former is president of the American Academy. The latter is the first surgeon who ever used ether in a capital operation, and has been for many years one of the surgeons of the hospital in Boston.

Leaving all this prolonged and usless discussion, the Committee next proceed, in great detail, to show that Dr. Jackson, after having made this induction, for four years, spoke of it to various persons, in the most public and decided manner. They then declare that, "*whether Morton had been before in pursuit of this object or not*, as he failed to find it till guided in the right way by a learned chemist, the judgment of mankind as to the chief merits of the discovery will be the same in either case." Why then, it may be asked, did the Committee think it important to make an elaborate effort to prove that Dr. Morton *had in fact made no prior experiment?* And here it is worthy of notice, that there is not the slightest attempt made to reconcile those fatal discrepancies, or to obviate those stringent objections which had been set forth in the majority Report, in its comments upon the witnesses in the case. Don Pedro Wilson and Dr. Keep here jog along together most harmoniously. The half dozen personal enemies of Dr. Morton, prove most satisfactorily that he unbosomed himself to them by admissions fatal to his own claims, and entirely at variance with his course publicly pursued with every body else, and on all other occasions—*and no attempt is made to explain this remarkable selection of confidants.** Mr. Metcalf, whose tes-

* In the course of the impeachment of Warren Hastings, he was enabled to prove himself of almost saint-like character, by the very people whom he had so inhumanly oppressed. By applying the thumb-screw to the poor inhabitants of Bengal, he extorted from their pain-quivering lips the confession of "good Lord," while their hearts silently cursed him with "good Devil," and this he triumphantly paraded before his peers as *evidence.* But confessions, even on the rack, cannot controvert the evidence of a man's actions. And the life, the daily wrong, the continued cruêlties and unabated wickedness of Warren Hastings, put the seal of silence and denial upon all evidence of this character, so that it signally recoiled upon him, almost to a summary ruin of his long protracted defense.

We find an analogy in the case before us. We care not whether it be the thumb-screw of fear and compulsion, or whether it is made of the less creditable materials of enmity and bitterness towards Dr. Morton, for having been discharged by him as unworthy of confidence, or for any other cause, its legitimate influence upon poor human nature, to the end, is the same. We have sometimes ventured to think it a little singular, that so much of Dr. Jackson's choicest testimony should emanate from the very quarter we are now contemplating.

All of the actions of Dr. Morton from the beginning to the consummation, every thing he has said or done, printed or written, all correspond with, and prophetically point to the great end accomplished.

Our readers are already familiar with the provoking discrepancy between the actions of Dr. Jackson and his claim. He has set about with remarktimony (from his known intelligence and high standing) is of the greatest importance to Dr. Morton, and who is able to fix dates, by the decisive circumstance of a voyage to Europe, is dismissed with the remark, that "his statement seems to be too vague to possess much weight, in view of so great a mass of conflicting testimony," and with some comments on the small size of the vial of ether which he saw in Dr. Morton's hands. Mr. Wightman's testimony (equally important and conclusive) the Committee endeavor to disparage, by intimating "that there is some extraordinary confusion in his dates;" carefully avoiding all allusion to those circumstances which render the *exact time* of his interview with Dr. Morton, *absolutely* and *demonstratively* certain, by written as well as *internal* evidence of the most satisfactory character.

The Committee in a former part of the Report had introduced in italics, and quoted with great emphasis, the statement of a new deponent, (Mr. Fowle,) who goes all lengths, and swears that Dr. J. told him in 1842, that by the use of ether "you can have a tooth extracted *or a limb cut off without pain.*" But it appears that Mr. Eddy, in 1846, had asked Dr. Jackson "whether he knew that the flesh of a person asleep from ether could be cut without pain," to which the able zeal to correct the great oversight; but we can only condole with him while we point to the *lateness of the hour.* It is in vain that he appeals to the buried years of the past to rise, that he may stealthily write modern truths on their records ere they sink again—unheeding, they slumber on. It is in vain that he refers to the utterance of familiar facts in science—they were printed long ago. It is in vain that his friends point to his reputation and ability, we will admit it all, but it does not help his case; for his actions are in advance of every thing, and they condemn him. "His case is hopeless, who, having nothing to say for his conduct, at length appeals to his character; the mercy of the court alone can save him."

reply was, "no; nor Morton either; he is a reckless man," &c. "the chance is he will kill somebody yet."

These two deponents (if their testimony had been brought together) would certainly seem to be somewhat at variance. The Committee resort to the adroit hypothesis, that Dr. J. merely meant, on the last occasion, to say that he did not *know* the fact—not to intimate that he did not fully *believe* it. The words actually used, it must be confessed, were oddly selected, even for the purpose of expressing belief.

In general it may be remarked that the tone of the whole document is unworthy of the Committee. It has not, in the least, a judicial character. It is an *argument for Dr. Jackson,* exactly such as *his* attorneys would be likely to have submitted, and which we really think must have emanated from that source, and have been adopted by the Committee, without any revision or modification. It throughout exaggerates the merit of the one, and depreciates that of the other party. Thus, it is not intimated that Sir Humphrey Davy* half a century ago suggested the same general idea of prevention of pain, in surgical operations—using, however, another agent, (nitrous oxyd;) that more than thirty years ago, a case was published to the medical world, of a man having been rendered lethargic by the use of *ether mixed with atmospheric air;* the effects produced being declared to be strikingly similar to those of nitrous oxyd and *also highly dangerous.* Not one word is said of Dr. Wells, of his experiments, conducted by means of the agent recommended by Davy—of Morton's knowledge of, and participation in, those experiments, (prior to his

* Davy's name is merely mentioned in Dr. Channing's letter.

purchase of sulphuric ether, sworn to by Metcalf) not one word of all this. The reader is left, instead, to infer that, to Dr. Jackson, Morton was indebted for the whole idea or conception. To *him* is given the concentrated credit due to the united genius and labors of all who preceded him—"a great truth was hidden," say the Committee, "and by *him* was first revealed."

It is not intimated, that during all those four years Dr. Jackson had never tried a single experiment for the purpose of demonstrating the *safety* of the agent employed, although it was, *as he well knew,* supposed by the profession, to be *extremely dangerous.* There is not the slightest mention of "the earnest or indefatigable labors of Dr. Morton," in bringing out this discovery, although we are told by one of the surgeons of the Hospital, "that he absolutely haunted them." On the other hand, not a word of comment is made on the preposterous claim of Dr. Jackson; that the *verification* of the discovery, no less than its *suggestion,* was wholly his.

There is not the slightest attempt to refute Dr. Edwards' demonstration upon this point. The minority, being unable to say any thing in favor of, and unwilling to say any thing against, Dr. Jackson, preserve, upon this branch of their inquiry, a discreet silence. *In other words, the whole Committee concur in according to Dr. Morton, the first actual application of ether.* We believe, indeed, that the only rebuke administered to Dr. J. by the Committee, is the very gentle one, of not being quite justified in becoming a party to the patent, "in violation of the recognized obligation of medical brotherhood."

Upon this subject of the patent, the same disingenuous course seems to have been adopted by the Committee. No attempt is made to reconcile with the "present *exclusive* claims of Dr. Jackson, the fact that he consented to become a joint patentee with Dr. Morton, and to receive only *one-tenth* part of its profits, and thereupon, even took an oath that they were joint discoverers."* Commenting severely on Dr. Morton's attempt to secure the patent for his own pecuniary benefit, no intimation is given of the formal attempt of Dr. Jackson (through his legal advisers) to obtain for himself, an increased share of its profits. While the Report heralds forth Dr. Morton's offer to *sell* his discovery to the government, for the use of the army and navy: and speaks of his attempt to "extort money from the nation's sufferings," it conceals his *gratuitous* offer of its use for both those departments, on account of the existing Mexican war—and his like *gratuitous* offer for

* "What would have been Dr. Jackson's position if he had merely permitted this discovery to go forth to the world as—in the specification accompanying the patent, he made oath that it really was—made jointly by himself—Dr. Morton? He has only, therefore, to remain silent, and he is sure of the chief honors of the discovery. The ribbon of France and the medal of Sweden will be his, and none may challenge his right to wear them. But, alas! he sees fit to claim all. Though all was virtually his before—and he realizes the fate of the dog in the fable, who "grasped the shadow and the substance missed." He, too, *opens his mouth,* and what fortune gave, folly lost. Dr. Jackson, must, indeed, as it seems to us, be classed among those who have been "ruined at their own request."

We speak advisedly when we say, that the *grand cross* of the legion of honor, has never been tendered to Dr. Jackson. He has simply received the offer of the rank of a "chevalier" of the legion of honor—that of a *private* in the *regiment*—being the lowest of four grades, while the grand cross is the highest. The honor tendered him was conferred so promiscuously by Louis Philippe, that it was on one occasion refused as being of a decidedly equivocal character. We have also been informed, on the authority of one who has seen it, that the *Swedish medal* makes no allusion to the ether discovery."

the benefit of every public charitable institution in the United States.

The paternity of this document (as the production of Dr. Jackson's attorneys) is rendered still more probable, by another trifling circumstance. In a pamphlet, entitled "Vindication of the Hospital Report of 1848," occurs the following paragraph:

"And here I will notice a slight, though unworthy, misrepresentation of the Messrs. Lord. They say, in speaking of the patent, 'Dr. Morton, *with most disinterested alacrity*, made it (the discovery, according to Mr. J. Bowditch) free as God's own sunshine;' whereas they well know that the Hospital Report denounces the patent in the strongest terms, and expresses the wish that it had been taken out rather from the hope of securing the *honor* than the *profits* of the discovery. What the Report says, is that the discovery '*had gone forth*, free as God's own sunshine;' and this, notwithstanding the patent." Now, this same misrepresentation re-appears in the minority Report, the expression in question being attributed not indeed to Mr. Bowditch by name, but to "one of the advocates of Dr. Morton."

The document closes with the following intelligence: "Note. Before the ink with which we penned our concluding sentence was dry, a telegraphic dispatch was laid before us, by Joseph L. Lord, Esq., of Boston, announcing, that on the 31st of January last, the *Institute of France* awarded the Cross of the Legion of Honor to Dr. Jackson, as the discoverer of etherization. It is extremely gratifying to find that our own views concur with the decision which has been pronounced in favor of Dr. Jackson, *by the most enlightened body of scientific men in the world.*

The Committee, unfortunately, were soon to be deprived of this gratification. Like other statements which they had made, on Mr. Lord's authority, this last—most flattering one—proves to have been *very highly colored.* Dr. Jackson had indeed received a *ribbon*—but the gift was in no wise connected with the *French Institute:* and he was himself, at last, obliged (when pressed by various newspaper inquiries) publicly to declare in print, *that he had never stated that there had been any formal decision of that body, in his favor:* and that the above announcement in the minority Report was the result of a *telegraphic mistake.* We believe, however, that he has never attempted to explain how it happened, that his attorneys, in his name and by his sanction, gave direct currency to the same "mistake" on various other public occasions, and *when no telegraph was used as the medium of communication.** We hardly think that Dr. Jackson will ever get a verdict in his favor from the French Academy. It seems certain, at any rate, that his claims are entirely overlooked by the American Academy, since that learned body has not even published, in its new volume, Dr. Jackson's communication, setting forth his pretensions as the discoverer of etherization.

A voluminous appendix accompanies this minority Report. The reader will find there all the affidavits in the case, which he has seen so often in former publications, and which must by this time have assumed a very familiar aspect. An unexpected discovery will,

*We are irresistibly reminded of another unfortunate *telegraphic mistake*, made several years ago, when he insisted upon relieving Prof. Morse of all the credit and annoyance connected with a certain world-renowned discovery.

however, reward his patient investigations, as he draws near the close of the pamphlet. He will there find various documents upon Dr. Morton's side, which (as it would seem) must have got into their present company by accident or inadvertence on the part of the Committee: There are the depositions of Spear and Leavitt,* upon which the majority Report had been led to speak of the "professional irregularity of one of Dr. Jackson's attorneys." A most important letter from Dr. Augustus A. Gould, of Boston, also, is there, charging those gentlemen with "a breach of courtesy and confidence"—exposing various inaccuracies in their statements, and expressly averring, that Dr. Morton, on an occasion alluded to, did mention to him his early ex-

*Fielding had an intimate knowledge of human nature. Tom Jones had been assailed by a jealous husband; and drawing his sword in self-defence, had inflicted on his opponent what was supposed to be a mortal wound. Blifil, an enemy of Jones, wishing to get him convicted of murder, by proving that *he struck first,* employs the professional services of counsellor Dowling to induce the witnesses of the transaction, to recollect this circumstance. The plot is suspected, and the lawyer is obliged to give a narrative of his agency in the matter. He says, "I told them, therefore, that if any offers should be made them, on the other side, *they should lose nothing* by being honest men, and *telling the truth.* I said *we were told that Mr. Jones had assaulted the gentleman, first,* and that, if that was the truth, they should declare it; and *I did give them some hints that they should be no losers.*" The lawyer, apparently having some slight misgivings, says, "I should not, I am sure, have proceeded such lengths for the sake of any other person living, but your worship." The reply is, "I think you *went lengths* indeed."

In exact accordance with this ancient precedent of Blifil *vs.* Jones, but with an entire unconsciousness of demerit, Mr. Lord, accompanied by a professional brother, waits on Mr. Leavitt, and says, "now Mr. Leavitt, what did you mean by swearing that I sought to bribe you." He replied, "you, or Mr. Lord, told me that *I should lose nothing* by signing such a statement; and *I supposed you meant to give me something,* if I would." Mr. Lord then asked Mr. Leavitt, if that was *all* the ground for saying he had bribed him. Mr. Leavitt replied in the affirmative.

periments, made before the interview with Jackson; adding, "*indeed I had many reasons for believing that experiments of the nature specified by him, had been performed.*" There is another most severe letter from Mr. Metcalf, charging Dr. Jackson's attorneys with "positive falsehood"—in which he says, "*Feeling now that Mr. Lord had no desire to understand what I really meant,*" &c. "*That Mr. Lord sought the interview for the purpose of catching me in some apparent contradiction, by cunningly devised questions, I have not now the least doubt; and failing to succeed as well as he had hoped, he resorts to the misrepresentations which I have pointed out,*" &c. &c. These latter documents throw great light upon the mode in which this controversy has been conducted on the part of Dr. Jackson and his friends. *None of them had before appeared in print: and verily we are astonished that Dr. Jackson and his friends should now wish to circulate them.* The Committee do not offer a word of explanation regarding these strictures which have fallen upon the Messrs. Lord, from so many distinct sources. An excellent letter also appears, addressed by Dr. Oliver W. Holmes (the poet-physician) to Hon. Isaac C. Morse, in which he says, "It is well known that Dr. Morton instead of profiting by his discovery, has suffered in mind, body and estate, in consequence of the time and toil he has consecrated to it."

"I have no particular relations with Dr. Morton, and no interest in common with him, to bias me in my opinion and feelings. But remembering what other countries have done for their public benefactors, and unwilling to believe that a rich and prosperous republic cannot afford, and will not incline to indulge its gratitude, whenever a proper occasion presents itself, I

have addressed you this line to tell you, that *I think now is the time, and this is the man.*"

Finally, a communication is published by which the high authority of Dr. James Jackson, of Boston, is claimed for Dr. Morton, whom he considers entitled to a grant from Congress for the "ether discovery, *more than any, and all other persons in the world.*" And we find also a letter from Dr. Henry J. Bigelow, of Boston, one of the surgeons of the Hospital, to Mr. Winthrop, which so *ably, clearly* and *concisely* states the *whole* argument, in favor of Morton, that we cannot refrain from quoting it entire.* Dr. B. is of the opinion of Paley, who says—"He alone *discovers* who *proves.*"

One letter laid before the Committee was treated with entire neglect; not being alluded to in either of

* Boston, January 26, 1848. Dear Sir: Learning that Dr. Morton is in Washington, and being much interested in the ether controversy, I take the liberty to write to you.

I believe most fully, that Dr. Morton deserves any reward Congress may grant to the discoverer; because, although many people have *thought* that a man could be intoxicated beyond the reach of pain, Dr. Morton alone *proved* this *previous possibility* to be a *certainty* and *safe.* A diagram will make the matter plainer than words:

| *Before* October, 1846. | Discovery in *Oct.* 1846. | *After* October, 1846. |
| --- | --- | --- |
| Who made the suggestion? Here is the only ground of dispute. | Consecutive experiments by Morton. | *Morton alone* took the responsibility of danger, and proved that ether was, 1st, *certain;* 2nd, *safe.* |

The two last points, viz. the consecutive experiments, and their confirmation, which *nobody disputes to Morton,* make him, in my eyes, the discoverer. The only doubt is, who made the *suggestion*? *To me, this is of no importance.* Dr. Jackson says, "I did. I told Mr. Morton to try the experiment; and unless I had so told him, he would never have tried it." Dr. Jackson adds, "I first tried ether when I was suffering from chlorine, in 1842. I afterwards recommended it to Mr. Peabody." But Dr. Morton confutes even these positions. He says to Dr. Jackson—1st, I show, by the evidence of Dr. Gould, Mr. Wightman and Mr. Metcalf, that I was experimenting with ether before the interview in which you the congressional reports. We refer to a long epistle from William T. Channing, of Boston, to a distinguished member of Congress. It takes up all the private and personal charges which have ever been circulated, in regard to Dr. Morton, and concludes by asking the Committee to investigate the truth of all of them before giving the sanction of Congress to one who had proved himself so undeserving. Finally, then, we would express our conviction, that the positions taken by Dr. Edwards are not in the slightest degree weakened by claim to have brought it to my notice. 2d, In 1842, you only re-discovered what was before clearly in print in Pereira's Materia Medica. 3rd, You claim to have told Mr. Peabody what you *knew* of ether. Now, you could not *know* it! You have stated all your grounds of deduction, and the widest inference you could draw from them, is a *suspicion* of the properties of ether, and a *suspicion* in science, an *unconfirmed theory*, amounts to nothing. Finally, what you claim to have discovered in 1842, you kept to yourself during four years. Do you expect the world to believe you knew its value? Do you expect it to reward you for letting people suffer during that length of time? Besides, the suggestion of anæsthetic agencies occurred to Davy; especially was it followed out, though unsuccessfully, by Horace Wells, who, disgusted with failure, abandoned his attempts. These and others had hypotheses, as well as Dr. Jackson. Morton alone proved the hypothesis. Without Morton, there is no evidence that the world would have known ether till the present day. I believe this covers the ground of important argument and difference in the pamphlets.

I beg you to allow for any inelegancies, resulting from my attempt at brevity, and to believe me, very truly and respectfully, your obedient friend and servant,

HENRY J. BIGELOW.

MR. WINTHROP.

There is not, probably, a more skillful surgeon in the United States than Dr. Bigelow. He has just been appointed Professor of Surgery in the Massachusetts Medical College, on the resignation of Dr. George Hayward. To a great power of imparting information orally, he unites a condensed style of writing. He apparently entertains the opinion, well expressed by a contemporary reviewer, respecting productions moderate in bulk and *portable*, viz. that "the light skiff will shoot the cataracts of time when a heavier vessel will infallibly go down."

any of the arguments in the minority Report: but that, on the contrary, they are confirmed by various new documents which, had they not been so unaccountably appended to that Report, would probably never have seen the light. The two Reports, indeed, as it seems to us, should be examined in the reversed order.

We believe that if any candid or unprejudiced person *after reading the minority Report* will take up that of Dr. Edwards, he will find it to be a complete *a priori reputation* of all that has since been so plausibly advanced in Dr. Jackson's behalf.

We sincerely congratulate Dr. Morton upon the fact that the opinion is constantly becoming more and more strong and general, that to *his* efforts and labors the world owes one of its choicest blessings. Though the honors already received by him have, through Dr. Jackson's instrumentality, been turned into insults,* and the

* The letter of Mr. Bowen, embodied in the minority Report, says, that Dr. Morton has had his reward "in a free gift of a thousand dollars, obtained from gentlemen, some of whom certainly imagined that they were subscribing only for the benevolent purpose of relieving him from debt: though, *with his usual unscrupulousness, he soon published their names as sanctioning his claim to the ether discovery, and they were compelled to disavow such an interpretation of their conduct.*"

What those gentlemen did *they were compelled to do,* not by their own feelings, *but by Dr. Jackson.* He, with a signal want of delicacy, addressed letters to the gentlemen who had united in this compliment to Dr. Morton, probably containing such representations as led them to suppose that Dr. Morton had made an improper use of their names. A communication from Dr. Jackson's attorneys was thereupon published in the Boston newspapers, with the replies from some of these subscribers. Among them was the following from Hon. Abbott Lawrence. "Park street, Oct. 21, 1848. My dear sir: In answer to your note, I beg to state that Mr. N. I. Bowditch called upon me some time within a year, and requested me to give a small sum of money for the relief of Dr. Morton, who was in great pecuniary distress. I gave most cheerfully the amount requested, because Mr. Bowditch desired me to do so.

compensation fairly his due from government, has, through the same instrumentality, been as yet withheld; we cannot doubt that his services will eventually obtain

"I have not the pleasure of an acquaintance with Dr. Morton, nor have I at any time authorized him to make use of my name in connection with the discovery of the application of ether. I am surprised that Dr. Morton should have used my name, as my only connection with him was *a mere act of charity,* such as are in our community occurring every day.

"I remain, dear sir, very faithfully, your obedient servant,

ABBOTT LAWRENCE.

"To Dr. C. T. JACKSON, Somerset street."

This publication drew forth a statement of facts from Mr. Bowditch, published in the Boston Transcript—who attributes Mr. L's letter to forgetfulness: stating that he *read* and signed the subscription paper, which was as follows: "In view of the benefit received by the public from the late ether discovery, and *with the desire of aiding towards the remuneration of Dr. Morton for his services and losses*—we, the subscribers, agree to pay the sums set against our respective names; the same to be applied by Samuel Frothingham and Thomas B. Curtis, Esqs. as they shall judge best for the benefit of Dr. Morton and his family."

Mr. B. then states that he informed Mr. Lawrence of the intention to raise $1000, in sums of $10, that the sum was raised and applied as intended—that a small surplus was invested in the box or casket, with inscriptions, which seemed warranted by the terms of the subscription paper—*that Mr. Lawrence, months before, had been apprised of all these facts, by a pamphlet published by Mr. Bowditch, and had never intimated any dissatisfaction, &c.*

And, subsequently, Dr. Morton addressed the following manly and courteous letter to Mr. Lawrence, published in the Boston Transcript of Dec. 12th:

Mr. Editor: A recent letter from the Hon. Abbott Lawrence having been published (doubtless with his permission) by the attorneys of Dr. C. T. Jackson, I was induced by what I felt to be a sense of self respect to return the ten dollars which he had subscribed for my benefit. As the note referred to appeared in your columns, I must ask of you the favor of inserting the following letter from me to Mr. Lawrence:

Boston, Dec. 6th, 1848, 19 Tremont Row. Hon. Abbott Lawrence, Sir: In May last I received from S. Frothingham and T. B. Curtis, Esqs. a silver box containing a subscription of one thousand dollars, to which was appended the names of more than one hundred of our most distinguished fellow citizens. Yours was among the number. I was, as I justly thought I might be, proud of such a testimonial, *which was wholly unsolicited on my part.* I accepted it gratefully as a voluntary gift from those a fitting reward, and that they will command the lasting gratitude of the country and of mankind.

It seems to us that these efforts of Dr. Jackson and his friends have signally failed. On the one hand, the CASKET is, in our opinion, something MORE than "a snuff box by way of charity," and, on the other hand, the RIBBON is something LESS than "a unanimous decision of the French Institute after a full sifting of all the evidence."

who wished "to aid in remunerating me for my services and losses" in the ether discovery.

As the subscription book was thus placed at my disposal, and the accompanying note of Messrs. Frothingham and Curtis expressed the kindest wishes in my behalf, I had no reason to doubt that the like sentiments were entertained by all the subscribers for whom they acted. In, therefore, printing an account of this donation and *with the names of the subscribers*, in an appendix to a little volume of mine on the teeth, *without any comment whatever*, and after the same account, without the names of the subscribers, had been already printed by others, I did not for a moment apprehend that I was taking a step contrary to the wishes or intentions of any of the donors.

A recent letter of yours, however, states that you gave the ten dollars in this case merely "as an act of private charity such as occurs every day," and that I had no authority whatever to use your name in any way in relation to the ether discovery. While I cheerfully acknowledge your well known liberality, both public and private, and feel grateful for your willingness *thus* to aid me, I yet feel, that, as I never solicited "private charity," nor authorized any one else to do so for me, I cannot retain the ten dollars, which you contributed, as I flattered myself, upon such different grounds. I therefore return the same. I would also express my regret at having unintentionally used your name in a manner which you consider unwarranted. Respectfully, yours,

W. T. G. MORTON.

Since this article was prepared, we learn that several of the young men who were interested in Dr. Jackson's favor, held appointments under him as U. S. Geologists; viz. Messrs. Wm. F. Channing, Barnes, McIntyre, and Peabody—a remarkable coincidence! We also learn that Messrs. J. W. Foster, of Ohio, and J. D. Whitney, of Massachusetts, have been recently appointed U. S. Geologists, to take charge of and complete the survey of the mineral lands, bordering on Lake Superior—being the survey which had been heretofore entrusted to Dr. Jackson. This change was made, after a full hearing before Mr. Secretary Ewing and Attorney General Johnson, and, notwithstanding, at that hearing, Dr. Jackson, among other incidental claims to consideration of the government, declared, "*I have annihilated human suffering.*"

